# Navigating Polypharmacy in Elderly Psychiatric Patients: A Review of the Literature

**DOI:** 10.1192/j.eurpsy.2025.1745

**Published:** 2025-08-26

**Authors:** R. A. Moreira, H. J. Gomes, E. Maldonado, I. Machado, J. M. Justo

**Affiliations:** 1 Psiquiatria, Unidade Local de Saúde do Nordeste, Bragança, Portugal

## Abstract

**Introduction:**

According to the World Health Organization, the global population aged 60 and older is projected to nearly double between 2015 and 2050, increasing from 12% to 22%. This demographic shift presents unique challenges in the management of psychopharmacological treatment in the elderly. Factors such as multimorbidity, atypical presentations of psychiatric disorders, age-related alterations in pharmacokinetics and pharmacodynamics, and the high prevalence of polypharmacy— often defined as the concomitant use of five or more medications—complicate therapeutic approaches in this population.

**Objectives:**

This review synthesizes the current evidence on the prevalence, contributing factors, and consequences of polypharmacy in elderly psychiatric patients.

**Methods:**

A narrative review was conducted using key terms and their combinations, including “polypharmacy”, “elderly”, “geriatric” and “psychiatry”. Relevant studies, case reports, and reviews published from 2000 to 2024 were included.

**Results:**

The prevalence of polypharmacy in the population varies across the literature; however, several well-established risk factors have been identified. These include age 65 years and older, living in a nursing home, comorbidities like diabetes mellitus, cardiovascular disease, metabolic syndrome, and COPD, as well as cognitive impairment. Additionally, prescription-related factors contribute to polypharmacy, such as poor quality of clinical records, automatic prescription renewals, and the involvement of multiple prescribers. The high rate of polypharmacy in older adults is associated with several challenges, including inappropriate medication use, difficulties in adhering to treatment regimens, increased risk of hospitalization, a higher likelihood of adverse effects, functional and cognitive decline, higher healthcare costs, and an increased risk of mortality. Additionally, a recent meta-analysis revealed for the first time that polypharmacy was significantly associated with the incidence of dementia and worsened its prognosis.

**Conclusions:**

Advanced age is a significant risk factor for polypharmacy, often resulting in adverse outcomes such as reduced quality of life, increased morbidity, and higher mortality rates. Identifying and regularly assessing polypharmacy cases, alongside evaluating potentially inappropriate medications, are critical steps. Implementing safe prescribing practices and deprescribing strategies specifically adapted to the needs of elderly patients can effectively reduce risks and improve overall well-being in this vulnerable population.

**Disclosure of Interest:**

None Declared

